# Neuroinflammation: An Integrating Overview of Reactive-Neuroimmune Cell Interactions in Health and Disease

**DOI:** 10.1155/2021/9999146

**Published:** 2021-05-31

**Authors:** Rodolfo Kölliker-Frers, Lucas Udovin, Matilde Otero-Losada, Tamara Kobiec, María Inés Herrera, Jorge Palacios, Gabriela Razzitte, Francisco Capani

**Affiliations:** ^1^Centro de Altos Estudios en Ciencias Humanas y de la Salud, Universidad Abierta Interamericana–Consejo Nacional de Investigaciones Científicas y Técnicas, CAECIHS, UAI-CONICET, Buenos Aires, Argentina; ^2^Hospital de Agudos JM Ramos Mejía, Buenos Aires, Argentina; ^3^Centro de Investigaciones en Psicología y Psicopedagogía (CIPP), Facultad de Psicología y Psicopedagogía, Pontificia Universidad Católica Argentina (UCA), Buenos Aires, Argentina; ^4^Instituto Universitario de Ciencias de la Salud, Fundación H.A Barceló, Buenos Aires, Argentina; ^5^Departamento de Biología, Universidad John F. Kennedy, Buenos Aires, Argentina; ^6^Facultad de Medicina, Universidad Autónoma de Chile, Santiago, Chile

## Abstract

The concept of central nervous system (CNS) inflammation has evolved over the last decades. Neuroinflammation is the response of reactive CNS components to altered homeostasis, regardless of the cause to be endogenous or exogenous. Neurological diseases, whether traumatic, neoplastic, ischemic, metabolic, toxic, infectious, autoimmune, developmental, or degenerative, involve direct and indirect immune-related neuroinflammation. Brain infiltrates of the innate and adaptive immune system cells appear in response to an infective or otherwise noxious agent and produce inflammatory mediators. Mediators of inflammation include local and recruited cells and signals. Processes derived from extrinsic and intrinsic CNS diseases also elicit the CNS inflammatory response. A deeper understanding of immune-related inflammation in health and disease is necessary to find potential therapeutic targets for preventing or reducing CNS damage. This review is aimed at discussing the innate and adaptive immune system functions and their roles in regulating brain cell responses in disease and homeostasis maintenance.

## 1. Introduction

The cardinal signs of acute inflammatory diseases involve cellular and molecular events, typically self-limiting, unlike autoimmune and neurodegenerative lesions, which are due to the failure in chronic inflammation resolution. Unresolved inflammatory conditions typically lack the proinflammatory to proresolving phase switch. This implies sustained recruitment and persistence of inflammatory cells at the site of inflammation because of lacking apoptosis and dead cell clearance, macrophages not switching to an anti-inflammatory/regenerative phenotype, no way out for the effector cells, and partial tissue regeneration.

Some of these unsuccessful resolution phase scenarios appear common to acute and chronic diseases.

Both in chronic inflammation with unsuccessful resolution and acute inflammation with a self-limited resolution, making sense of the interaction interlayer between parenchyma cells and immune cells is key to understanding the inflammation-repair process.

Both the immune and central nervous (CNS) systems produce and use immune factors and neuroendocrine mediators. Immune cells and mediators play a regulatory role in the CNS, participating in neurodevelopmental synaptic plasticity and removal and synaptic plasticity in adulthood. Far-distance talk of immune cells with the CNS allows the immune system to engage the body in fighting infection by pathogenic microorganisms and the nervous system to regulate immunity.

Cross-talk between the immune, nervous, and endocrine systems involves a great variety of mediators, including cytokines, neurotransmitters, and hormones.

The nervous system lays out functional connections with the immune system directly innervating the lymphoid system with adrenergic, peptidergic, and catecholaminergic fibers and via receptors for neuropeptides (substance P (SP), somatostatin, and vasointestinal peptide, (VIP)) and neurotransmitters (noradrenaline, acetylcholine, enkephalin, and endorphin) on immune cells. These mediators can modulate the synthesis and release of cytokines, including the chemokines, chemotactic cytokines. Chemokines are low molecular weight cytokines that recruit secondary proinflammatory leukocytes and might act as central neuromodulators [1]. Neuropeptides and neurotransmitters reach the immune cells by nerve-terminal nearby diffusion (nonsynaptic transmission) and bloodstream circulation. Hormonal receptors in lymphoid tissues allow the neuroendocrine mediators to interact with the immune system. In the last years, the consistent characterization of receptors and hormones in lymphoid tissues has brought out interesting information on the cross-talk between the immune and neuroendocrine systems and the involved mechanisms.

Brain tissue is a particular target of immune-inflammatory reactions. In the past, the CNS was believed immune-privileged, v.g., not prone to undergo strong inflammation, and lacking lymphatic drainage. Accrued evidence on neuroimmune interactions has questioned the historical idea of the brain, isolated by the blood-brain barrier (BBB), immune-privileged. This intrinsic characteristic of the CNS is conferred by constitutive and reactive components including the BBB, microglial cells, astrocytes, oligodendrocytes, and infiltrating myeloid and lymphoid cells. Astrocytes appear to protect the CNS from T cell-mediated neuroinflammation [2]. This review offers an update on the key inflammatory mediators and the role of inflammatory cells in infectious and noninfectious conditions on neuroinflammation. We discuss the relationship between neuroinflammatory processes, hypoxia, and oxidative stress and how innate and adaptive immunity shape up an integrative network to regulate immunological processes, affecting brain homeostasis.

## 2. Neuroinflammatory Diseases

The BBB-derived immune privilege of the brain is, at least, questionable by now. Central nervous system cells are reactive to peripheral inflammatory factors, and peripheral immune cells can infiltrate the brain. In encephalitis, meningitis, encephalopathy, hypoxia, and other conditions, the inflammatory response of brain cells evidences neurological involvement. Neurologic manifestations of infective (parasite, virus, bacteria, and fungi) and not mutually exclusive, noninfective agents (traumatic, neurodegenerative, and autoimmune) result in morbidity and mortality. The best treatment for these neurologic complications, with varying degrees of recovery and sequelae, is yet unclear.

Inflammation is emerging as a pivotal mechanism common to different neuropathological conditions [3–10].

### 2.1. Neuroinfectious Diseases

Innate immunity offers a rapid response to infections, often called the first line of host defense, enhancing adaptive immune responses. During neuroinflammatory infections, specific types of innate immune molecular and cell pathways seem activated. Their functional effectiveness to limit brain injury spread is crucial.

Neurologic dysfunction with acute alteration in mental status due to inflammation is a hallmark of CNS infections by neurotropic pathogens [11]. Postinfectious neurologic dysfunction has been attributed to irreversible damage caused by pathogens on their own [12–15].

Neurologic involvement and manifestations were reported in some parasitic infections, v.g., Chagas disease, toxoplasmosis, human African and American trypanosomiasis, echinococcosis, cysticercosis, leishmaniasis, onchocerciasis, schistosomiasis, food-borne trematodiasis, dracunculiasis, filariasis, and soil-transmitted helminthiasis [16].

Chagas disease is associated with brain atrophy independent from structural cardiac disease related to cardiomyopathy. Brain atrophy, rather than multiple infarcts, may represent the main anatomical substrate of cognitive impairment in Chagas' disease [17].

An important determinant of brain inflammation is the delicate balance between proinflammatory and counterinflammatory mediators. In mouse models of human African trypanosomiasis, proinflammatory mediators like the tumor necrosis factor (TNF-*α*), interferon-gamma (IFN-*γ*), and CXC ligand 10 (CXCL10) have been crucial to parasite CNS invasion. The administration of IL-10, a prototypical counterinflammatory molecule, reduces the CNS parasite burden, the severity of the neuroinflammatory response, and the clinical symptoms associated [18].

Viral infections associated, or not, with acquired immunodeficiency like dengue, rabies, infections by Epstein Barr virus (EBV), herpes papillomavirus (HPV), human immunodeficiency virus (HIV), and others could cause neurological complications.

Human immunodeficiency viruses infect the CNS during primary infection and persist in resident macrophages, leading to low-grade chronic inflammation. Various CNS viral infection-mediated inflammations take place in perivascular inflammatory infiltrates of the CNS parenchyma [19].

Malignant and nonmalignant tumors are rare. Based on serologic findings and literature, the pathogenetic mechanism of this rare intracranial tumor is believed a chronic reactive response to EBV infection [20].

Bacterial infections of the CNS can cause meningitis, granulomatous infections like tuberculosis, syphilis, spirochete infections, and others, cerebral and epidural abscesses, and bacterial exotoxin-related diseases like diphtheria, tetanus, and botulism, affecting the CNS.

Certain mycoses can affect the brain causing neuroinflammation and neurodegeneration [5, 7, 8] or toxicity [21]. Coccidioidal meningitis (CM) often affects immunocompromised people [22]. Cerebral aspergillosis is a highly fatal infection [23], and mucormycosis is an opportunistic fungal infection with a poor prognosis among generalized fungal infections that promote brain degeneration.

The past years have established a key role for infectious pathogens in certain neurological autoimmune-associated diseases. Certain systemic and organ-specific autoimmune diseases, rheumatic mainly, can cause neuroinflammation. Fibromyalgia [4], destructive joint diseases [6], and systemic lupus erythematosus [3] are a few examples. Neurodegeneration studies suggest that peripheral infection might be related to onset and progression of age-related neurodegeneration [24]. Aged patients appear more vulnerable to infection-related cognitive changes associated with Alzheimer's disease (AD). This may occur from typical infectious challenges like respiratory tract infections, although some specific viral, bacterial, and fungal pathogens have been associated with disease development as well. To date, whether these microorganisms are directly related to AD progression or are opportunistic pathogens colonizing dementia patients and exacerbating the preexisting ongoing inflammation [8, 25] is unclear.

Neuroinflammation with altered synaptic plasticity following perinatal infectious-inflammatory challenges is of concern. The effects of congenital infection on neural cell proliferation and survival, axonal damage, and myelination have been studied in different experimental settings [26]. Microglia, as an antigen-presenting cell (APC), exerts a special role during neuroinflammation-associated injury to the immature brain [27]. However, microglial activation also participates in the immunoregulation-triggering response, although the evidence suggests that the microglia critically influences brain plasticity in the healthy developing brain [28].

### 2.2. Not Pathogen-Associated CNS Diseases

Neurodegenerative diseases progressively affect cognitive and motor functions and interfere with daily tasks' performance. Advances in genetics and animal models are showing an unexpected role of the immune system in the pathogenesis and onset of diseases. The role of cytokines, growth factors, and immune signaling pathways in disease pathogenesis is still being examined [29].

Traumatic brain injury (TBI) elicits a robust immune response within hours and days [30]. Peripheral immune cell infiltration to the damaged tissue with activation of brain resident astrocytes and microglia has been observed in patients and TBI animal models. Regulatory T cell-reduced neuroinflammation, T lymphocyte brain infiltration, reactive astrogliosis, interferon-*γ* gene expression, and transient motor deficits have been observed in an acute TBI murine model [31].

Postmortem brain and cerebrospinal fluid of Parkinson's disease (PD) patients had a high concentration of proinflammatory cytokines, indicating ongoing neuroinflammation beyond pathology. Inflammation might lead to oxidative stress promoting dopaminergic neuron degeneration [9]. Several studies have reported inflammation and immune responses as determinant factors in disease progression, responsible for pathogenic processes in familial and sporadic PD onset [10]. One study reported activated microglia in the substantia nigra (SN) and putamen of patients diagnosed with PD [32]. In 2005, another study suggested microglia-mediated inflammation presenting at an early stage of parkinsonism [33]. Several authors suggested pathogenic mutations in the *α*-synuclein (SNCA) gene and the leucine-rich repeat kinase 2 (LRRK2). Alpha-synuclein accumulation, a major stimulant of microglial activation, participates in PD progression [34–36]. Both central and peripheral inflammation is responsible for the sustained progression of PD. Degeneration of dopaminergic neurons occurs with the infiltration of T cells and activation of microglia, with increased production of inflammatory cytokines and chemokines due to pathological SNCA accumulation [34, 37, 38].

In addition, the CNS is an autoimmune disease target. Multiple sclerosis (MS) is one of the most ravaging disorders, presenting with spontaneous onset, remitting-relapsing periods sometimes, and a progressive disease pattern in genetically predisposed hosts. Experimental autoimmune encephalomyelitis (EAE) is the traditional animal model for MS. However, despite its similarities with MS, most treatments for EAE have failed in translation to humans. Adaptive and innate, systemic, and resident in the CNS immune components contribute to neurodegenerative and neurobehavioral disorders' progression as found in animal models and correlated with human studies.

Environmental triggers affecting the CNS during the prenatal and postnatal periods trigger microglia activation and astrogliosis, upregulate proinflammatory cytokines, and are critically associated with neuroinflammation [39, 40]. It is not only a hallmark of infections but secondary to not-infective insults as well, like cerebral hypoxia-ischemia [41]. Noteworthily, inflammatory brain glial cells appear pivotal in regulating synaptic structure and function. Synaptic physiology and pathophysiology studies suggest that the immune system dynamically affects neurodevelopmental synapse organization [42, 43].

Though seldom exposed to harmful agents, brain tissue has limited restorative ability to repair damaged cells. The expanding molecular biology findings offer increasing insights into immune glial system interactions, including innate and adaptive immune molecules and receptors mediating tissue injury and repair [44].

## 3. Key Components of the Neuroinflammatory Process

Newly evolving neuropathology evidence offers proper interpretations of a plethora of diverse disorders. Microglia response, infiltrating immune cells, generation of oxidative stress species, and proinflammatory cytokines offer a common background to neuroinflammatory and neuroimmune responses.

Neuroinflammation is often harmful yet contributes to normal brain development [42] and homeostasis and is actually necessary for brain plasticity during critical developmental periods [45]. Perpetuating inflammatory processes lead to progressive chronic inflammatory conditions, mainly derived from autoimmune or neurodegenerative disorders. Adaptive immune-mediated neuroinflammation is a frontier grey zone between injury and healing in chronic diseases in particular.

In homeostasis, the neuronal function requires glial cells and BBB integrity. Accumulating evidence suggests that neuroinflammation targeting glial cells is implicated in neurodegenerative disorders [46].

### 3.1. Inflammatory Mediators in the CNS

#### 3.1.1. Cytokines and Chemokines at the Neuroinflammation Border

Chemokines and cytokines are bioactive proteins and peptides involved in feedback activation of protein signaling cascades. Peripheral macrophages and lymphocytes and central astrocytes and microglia produce and release cytokines and chemokines. These are necessary for neuronal metabolism, immune surveillance, leukocyte trafficking, and uptake of other inflammatory mediators. They participate in neurodevelopment and synaptic transmission and are the main inducers of neuroinflammation. Cytokines and chemokines bind to specific membrane receptors at the extracellular ligand-binding region, activating the intracellular region which triggers signal transmission to the nucleus [47].

Cytokines and chemokines are neuroprotective and neuroinflammatory, and their dysregulation is decisive for neuroinflammation, neurodegeneration, and demyelination in the central and peripheral nervous systems [48].

*(1) Chemokines*. Chemokines comprise two categories based on their expression. One of them, constitutively expressed, is responsible for the maintenance of homeostasis, surveillance, and immune system monitoring. The other one, inducible by inflammation following damage, amplifies the innate and adaptive immune system responses.

Chemokines act via chemokine-unspecific G protein-coupled receptors (GPCR). They can attract or activate immune cells and affect neuronal activity and survival [49]. They may induce neuronal death directly, activating neuronal chemokine receptors, or indirectly, activating microglial killing mechanisms. Some chemokines are neuroprotective and act as pro- or anti-inflammatory mediators [48]. One of the most important neuroinflammatory chemokines is the monocyte chemoattractant protein-1 (MCP-1), also known as C-C motif ligand 2 (CCL2) or C-X3-C motif ligand 1 (CX3CL1). It regulates the migration of monocytes, T lymphocytes, and “natural killer” cells towards the affected area. In its soluble form, MCP-1 participates in the interaction between neurons and other inflammatory cells.

The MCP-1 acts via the CCR2 receptor and is expressed in neurons and glial cells. The astrocytes are the major source of MCP-1 after neuronal damage or infection. It plays an important role in neuroinflammation linked to various diseases involving neuronal degeneration. Neuronal MCP-1/CCL2 induction during mild impairment of oxidative metabolism caused by microglial recruitment/activation exacerbated neurodegeneration in thiamine deficiency- (TD-) induced neuronal death. Knockout mice lacking CCL2 were resistant to TD-induced neuronal death, suggesting that CCL2 mediated microglial recruitment and neurodegeneration in this model [50]. However, several studies show that suppressing MCP-1 may be beneficial, reducing inflammation in some diseases. In patients with complications associated with inflammatory processes, a high blood level of CCL2 contributes to ischemic cerebrovascular disease and myocardium infarct. Brain overexpression of CCL2 aggravates ischemic injury [51], while CCL2 deficiency confers neuroprotection against permanent carotid artery obliteration [52]. Mice lacking CCR2 showed reduced cerebral edema, infarct size, and BBB disruption and decreased leukocyte, monocytes, and neutrophil infiltration. They also had decreased expression of a variety of proinflammatory cytokines (IL-1*β*, TNF-*α*, and IFN-*γ*) and endothelial cell adhesion molecules preventing leukocyte-endothelial cell interaction during reperfusion [53, 54]. Interestingly, MCP-1-deficient mice showed reduced neuroinflammatory responses and increased peripheral inflammatory responses to peripheral endotoxin insult [55]. In the hypoxia-ischemia model, CCR2 knockout mice had impeded transendothelial diapedesis in response to CCL2, showing that CCR2 was required for stem cell migration to promote CNS regeneration via CCL2 chemotaxis [56]. Likewise, CCL2 protected cultures of human neurons and astrocytes from glutamate toxicity and HIV-transactivator of transcription- (HIV-tat-) induced apoptosis [57]. Rat dorsal hippocampal neurons in culture treated with kainic acid (KA) showed increased CCL2 and macrophage inflammatory protein-2 (MIP-2) levels, both inducers of basic fibroblast growth factor (bFGF) and astrocyte activation. Astrocytes stimulated with CCL2 facilitated bFGF-dependent neuronal cell differentiation and induced H19-7 neurons' survival in vitro, suggesting a supporting trophic role for chemokine-activated astrocytes [58]. Astrocytes produce chemokines in response to proinflammatory cytokines like IL-1*β* and TNF-*α* and synthesize MCP-1 via nuclear factor-kappa B (NF-*κ*B) [55]. Primary astrocytes treated with lipopolysaccharide (LPS) and interleukin- (IL-) 1*β* were responsible for the exacerbated cytokine response observed in vivo in the absence of CCL2 postinjury. Evidence of CCL2-induced inhibition of IL-6 and TNF-*α* produced by astrocytes following IL-1*β* stimulation suggests a novel CCL2 immunomodulatory role in acute neuroinflammation [59].

Other chemokines like CXCL9, CXCL10, and CXCL11 and their receptor (CXCR3) are crucially involved in AD and MS. They are implicated in the Th1-type response in various diseases. Their expression is induced by IFN-*γ*, the most typical Th1 cytokine associated with tissue T cell infiltration [60]. Accordingly, MCP-1 might have a dual neuroinflammatory or neuroprotective role in neurodegenerative diseases, depending on the neuroinflammatory milieu.

*(2) Cytokines*. The small proteins known as cytokines are signaling molecules released in response to a variety of stimuli under physiological and pathological conditions. Present in up to picomolar concentrations, they regulate inflammation and the duration of the immune response and modulate cellular activities like growth, survival, and differentiation. The large and diversified group of pro- or anti-inflammatory cytokines comprises different families based on their structural homology and that of their receptors [48]. The main proinflammatory cytokines are TNF-*α*, IL-1*β*, and IL-6 interleukins and IFNs. Anti-inflammatory cytokines are IL-10 and IL-4, among others. Cytokines act as neuromodulators and regulate neurodevelopment, neuroinflammation, and synaptic transmission. They are crucial to brain immunity comaintaining immune surveillance, facilitating leukocyte traffic, and recruiting other inflammatory factors. The role of cytokines in neurodegenerative diseases is complicated by their dual roles in neuroprotection and neurodegeneration [48].

To illustrate, IL-6 has dual roles in brain injury and disease. It is essential in regulating inflammation, balancing between pro- and anti-inflammatory responses, and participating in neurodegenerative and neuroprotective processes [61]. The peripheral nervous system and the CNS, v.g., neurons, microglia, and astrocytes, in particular, show IL-6 [60]. In neuroinflammatory processes, IL-6 promotes astrogliosis and microglial activation. During reactive astrogliosis, IL-6 acts as a neurotrophin, promoting neuronal survival in response to neuronal damage. A high level of IL-6 has been associated with brain disease [61]. Interleukin-6 is upregulated upon neuroinflammation as observed after CNS infection or injury, viral meningitis, experimental encephalitis, and acute viral infections. In all these conditions, its cerebrospinal fluid (CSF) level rises in patients [62]. Other examples of high IL-6 level conditions are mouse experimental cerebral malaria [63], TBI [64], and advanced stages of patients with HIV infection [65]. Conversely, studies in IL-6 knockout mice show a compromised inflammatory response, increased oxidative stress, impaired neuroglial activation, decreased lymphocyte recruitment, and a slower rate of recovery and healing [61]. In physiological conditions, TNF-*α* participates in homeostasis regulation, synaptic plasticity, learning and memory, and sleep/wake cycles. However, a high TNF-*α* level is related to neuroinflammation and neurodegenerative diseases [66]. Its major source is the microglia, along with astrocytes and neurons during neuroinflammation [66]. Together with the interferon-gamma protein (IFN-*γ*), TNF-*α* is proinflammatory during acute brain inflammation and is immunosuppressive upon chronicity [67].

Interferon-gamma (IFN-*γ*) is a multifunctional cytokine that participates in inflammation onset and consolidation, in innate and adaptive immune responses, induced in many cell types, including neurons [68]. It is a potent inducer of TNF-*α* gene expression in microglia, having complementary roles during neuroinflammation [66]. TNF-*α* induces neurotoxicity by high glutamate production, leading to neuronal excitotoxicity and death [69]. Inactivating IL-1𝛽 and TNF-*α* with neutralizing antibodies reduced neuronal death in SK-N-SH cells, a neuroblastoma cell line induced by the West Nile virus [70]. Deleting the TNF-*α* gene reduces neurodegeneration in Sandhoff disease (SD), a lysosomal storage disorder [71]. However, TNF-*α* receptor-1-deficient mice showed severe experimental autoimmune neuritis suggesting an anti-inflammatory role for TNF-*α* at least in this model [72]. Two surface receptors, TNFR1 and TNFR2, recognize TNF-*α*. They differ in their expression, signaling cascade transduction, and TNF-*α* binding affinity [73]. Downregulating TNFR1 reduced JNK activation and attenuated neuroinflammation, neurovascular damage, and brain injury in the LPS-sensitized hypoxic-ischemia mouse model [69]. Upregulating TNFR2 protected neurons from excitotoxicity and promoted neuronal survival, activating the PI3K/NF-*κ*B signaling pathway in a glutamate-induced cell death model [74]. Different receptor-related signaling pathways account for TNF-*α* dual effects [75, 76].

IL-1*β* is a very potent signaling molecule of the family of pleiotropic cytokines, expressed at low levels usually, but induced rapidly in response to local or peripheral insults. It coordinates the host defense response to pathogens and injury, not surprisingly, not only systemically but in the CNS as well. Upon injury or in brain disease, IL-1*β* presence has been correlated with effects on neurons and nonneuronal cells [77]. It is also involved in neuroinflammation, fever, appetite, learning, and memory [78]. It is synthesized by macrophages, microglia, astrocytes, T and B lymphocytes, or neutrophils, among others [77]. Binding to the IL-1R receptor induces the production of other inflammatory cytokines like IL-6 and TNF-*α*, and the increase in the PLA2, COX-2, and iNOS enzymes which produce arachidonic acid, prostaglandins, and NO, respectively [79, 80]. Studies in IL-1R1 receptor-deficient mice found decreased activation of microglia and astrocytes and of IL-6 and COX-2 production in brain injury, indicating the key role of IL-1*β* [79, 81]. Interleukin-1*β* was rapidly induced in experimental stroke, while a low IL-1*β* level protected from ischemic injury and neuronal loss, reducing infarct volume [79, 82]. Multiple sclerosis patients had high IL-1*β* levels in CSF and demyelinated lesions [83]. Oppositely, IL-1*β* induced the production of fibroblast growth factor-2 (FGF-2), transforming growth factor-*β*1 (TGF-*β*1), and nerve growth factor (NGF), promoting neurite growth in vitro [84]. Taken together, IL-1*β* appears important in the initiation and development of the inflammatory cascade and in neuronal survival in a variety of neurodegenerative diseases.

Neuropoietic cytokines are a group of immune mediators that participate in normal brain development, promoting neural precursors' proliferation, fate determination and differentiation, neuronal and glia migration, cell survival, and activity-dependent changes in synaptic function. Inflammation during development may cause widespread injury, interfering with the normal balance in cytokine signaling and developmental processes, or increase neurological vulnerability later in life [85].

#### 3.1.2. The Role of the Complement Cascade in Neuroinflammation

The complement system comprises around 30 proteins, nearly 5% of total whey protein and a low proportion of membrane proteins. It participates in the recognition, trafficking, and elimination of pathogens and any unfamiliar material to the host as a powerful arm of the innate immune system. In normal conditions, its components do not pass through the blood-brain barrier. Glial cells and neurons produce complement components, largely in response to neural damage or inflammatory signals [86]. The complement cascade is also expressed during physiological development when neuron-derived complement proteins tag synapses for pruning by microglial cells [87, 88]. Astrocytes and microglia are the largest producers of complement elements in both normal and pathological conditions. The microglia expresses high complement receptor levels, crucial at inducing phagocytosis of complement-labeled structures, regulating cytokine signals and chemotaxis. Astrocytes, oligodendrocytes, and neurons express high levels of the C3 complement fraction and other members of the complement cascade [89, 90]. The complement system is implicated in several neurological disorders. Complement mRNAs have been found in the cerebral cortex and the hippocampus in man. The postmortem examination of samples from patients with AD showed an increased level of these mRNAs in pyramidal neurons. This finding along with reactive oxygen species and proteases portrays a local inflammatory nest compatible with neuronal dysfunction and cognitive decline [91–93]. Products of the activation cascades are generated in human AD, MS, Huntington's disease, Parkinson's disease, spinal cord injury, TBI, and cerebral ischemia [94–102]. In addition, C3^−/−^ mice showed reduced brain edema in intracerebral hemorrhage [102]. Complement overactivation, associated with glial activation and the release of proinflammatory compounds, appears implicated in synaptic loss concomitant with aging physiological, cognitive decline, and brain diseases [103]. The complement system role in the pathology of neurodegenerative diseases opens new avenues for understanding its involvement in neuroinflammatory processes and as a promising target for future therapeutic strategies.

### 3.2. Inflammatory Cells in the CNS

Neurons and glial cells produce cytokines either constitutively or by induction in appropriate culture media.

Glial cells, unlike neurons, are not excitable and comprise the microglia and the macroglia (astrocytes, oligodendrocytes, and ependymal cells). Some of them are involved in the isolation, support, and supply of substances to maintain neuronal metabolism. The microglia are considered brain resident macrophages able to migrate to the inflammatory foci. Glial cells release cytokines, which establish functional connections with each other and with neurons. Upon inflammatory stimuli, they can participate in the pathogenesis of neurological diseases.

#### 3.2.1. Microglia

Microglia, the resident immune cells of the CNS, are derived from yolk sac macrophages arising during the first wave of primitive hematopoiesis and populating the developing CNS via the bloodstream once embryonic circulation is established [104]. Central nervous system glia and a mononuclear phagocyte are involved in physiologic processes, inflammatory and immune responses, and in the pathogenesis of several CNS disorders [46]. These cells share innate immunological functions with other mononuclear phagocytes like monocytes, macrophages, and dendritic cells, mostly related to phenotypic characteristics and lineage-related immunological properties, including the ability to secrete cytokines common to immune antigen-presenting cells, described over two decades ago [105].

Surveillant microglia cells contribute to maintaining CNS homeostasis [106]. In response to inflammation challenge, microglia promptly becomes ameboid and upregulates cell surface receptors involved in innate immune responses, proinflammatory type (classical or M1 activation). This is because they have pattern recognition receptors (PRRs) like the toll-like receptors (TLR), the nucleotide-binding oligomerization domain-like (NOD) receptors, receptors for advanced glycation end products (RAGE), scavenger receptors (CD36, CD91), phagocytic receptors like the CR3 and CR4, and triggering receptor expressed on myeloid cells (TREM). These receptors are involved in the innate immune response, increasing the expression of various cytokines, chemokines, surface receptors, and metabolic enzymes [107]. The microglia can take on an anti-inflammatory profile (M2 microglia), promoting healing, tissue regeneration, and angiogenesis. The M2 microglia has been subdivided into different M2 subtypes depending on the expression of specific markers and secreted cytokines and chemokines [82, 108].

Microglia is crucial in restricting neuroinflammation. In osteopetrotic (op/op) mice, defective in producing functional colony-stimulating factor (M-CSF), a decrease in the number of tissue macrophages and microglial cells led to neuropathology exacerbation [109, 110]. All the same, resident glial cells can turn into aggressive effectors, attacking healthy neurons by phagocytosis, or secreting factors on their own, or in coordination with infiltrated immune cells [111]. This rich repertoire of responses may account for the dichotomic microglia reactivity in promoting neuronal survival or degeneration.

The presence of activated microglia in nearly every neurological insult leads to possibly oversimplifying in vitro study design. Assuming that activated microglia and associated inflammatory responses are harmful to the brain should be cautious [112]. The reactive response of the microglia might be interpreted mostly as beneficial. The regulatory control of neuroinflammation is normally imposed, and interfering with homeostatic regulations may be detrimental. Unfortunately, the way to reaching a healthy balance and its modulation under psychological distress and neurological diseases is still unclear [107].

#### 3.2.2. Astrocytes

Astrocytes have been traditionally considered supportive cells for neurons, responsible for brain homeostasis and neuronal functions. They are the largest cell population in the CNS, even compared with neurons [107]. Astrocytes give metabolic support to the neuron, generate neurovascular coupling, and control BBB permeability. They are essential in recapturing several neurotransmitters, K^+^ damping, and other functions. They express a wide variety of cytokine receptors like the PRRs, contributing to brain immunity [113]. The expression kinetics indicates that chemokines contribute to amplifying the inflammatory reaction or that astrocytes can promote recruitment and proliferation of regulatory T cells (Tregs) via the anti-inflammatory cytokine transforming growth factor *β* (TGF-*β*) and chemokine CXCL12 (stromal cell-derived factor-1 (SDF-1)) [107]. Astrocytes secreting other anti-inflammatory cytokines like IL-10 might exert important immunoregulatory functions in the CNS, reducing microglia and astrocytes' presenting capacity and interfering with antigen-specific T lymphocyte proliferation.

Activated T lymphocytes (Th1 and Th17) in the secondary lymphatic organs cross the BBB and are locally reactivated upon surface antigen recognition on the antigen-presenting cells. They secrete cytokines that stimulate microglia and astrocytes, increasing cell recruitment in a variety of neurological disorders. Astrocytes act as a source of cell surface receptor/ligands and cytokines to modulate both innate and adaptive immune cell system in the neuropathy, and the way around, immune cells regulate astrocyte activity [114, 115].

Microglia and astrocytes play an active dual role in brain inflammatory diseases. Not only can they boost immune responses and promote neurodegeneration but can also protect and restrict CNS inflammation. What factors or scenarios determine whether a beneficial or detrimental response follows remains a matter of research.

#### 3.2.3. Oligodendrocytes

The oligodendrocytes are glial cells that start myelinization, allow electric potential propagation, and give metabolic support to neurons. From an immunological point of view, oligodendrocytes were classically thought of as inert and merely representing bystander victims of immune responses. This view has now changed in the light of accumulating evidence that oligodendrocytes actively produce a wide range of immune-regulatory factors and express the corresponding receptors [115].

Neuroinflammatory responses can be deleterious for cell survival, leading to irreversible and extensive brain damage, if long-sustained in particular. Oligodendrocytes are the main target of the immunoinflammatory response in the CNS. This occurs due to deleterious cytokines released by infiltrating macrophages and microglia, T lymphocyte cytotoxicity, or antibodies triggering antibody-mediated cytotoxicity (antibody-dependent cellular cytotoxicity).

Oligodendrocytes produce immune mediators that modulate microglia activity in response to stress. Chemoattractants like CXCL10, CCL2, CXCR2, and CCL3, CXCR2 expressed on oligodendroglia, in particular, have been implicated in the pathogenesis of neuroinflammatory demyelinating diseases and in amplifying the migration, proliferation, and myelin production by the oligodendroglia [116]. Oligodendrocytes express receptors to IL-4, IL-6, IL-10, IL-12, and other cytokines and markers like the CD200 during inflammation and infection, suggesting that they recruit microglia to damaged tissues [115]. A wide range of proinflammatory cytokines, including IL 1, 2, and 3, IFN *α*, *β*, and *γ*, TNF-*α*, and lymphotoxin, released by microglia, have been detected in demyelinating pathologies like MS, suggesting that microglial activity and oligodendrocyte damage may be associated [116, 117]. In vitro stimulation with IFN-*γ* induced MHC-I expression, making them susceptible to death caused by CD8+ T cells (often called cytotoxic T lymphocytes) [118]. Likewise, oligodendrocytes express both IL-18 and IL-18R receptors during the active MS period. The large amount of IFN-*γ* observed in these circumstances adds to oligodendrocyte damage [119]. Human oligodendrocytes are susceptible to MHC class I restricted CD8+ T cell mediated cytotoxicity in vitro [120, 121], to non-MHC restricted cytotoxicity mediated by *γδ* T cells [122], and to cytokine-activated natural killer (NK) cells [123]. The cytotoxic activity of killer (K) cells in enriched cultures of bovine oligodendrocytes (BOL) was investigated in MS. Human K cells mediated cytotoxicity to primary cultures of BOL, where the antibody-dependent cell-mediated cytotoxicity (ADCC) to BOL was mediated by large granular lymphocytes [124].

**Figure 1:**
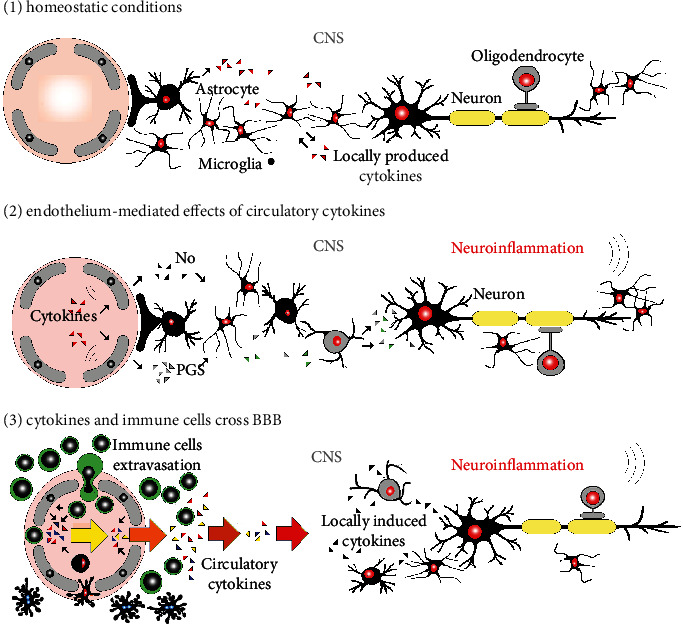
Essential ways cytokines affect the brain. (1) De novo synthesis of cytokines in the CNS in homeostatic conditions is clear. (2) Peripheral cytokines can induce brain cytokine synthesis. Also, cytokines can act centrally via endothelial cells. Cytokine-endothelial cell interaction triggers the release of second messengers like nitric oxide (NO) and prostaglandins (PGS) with central effects. Hence, the signal mediated by a cytokine as IL-1*β* can be transduced from the periphery without crossing the BBB. (3) Systemic administration of IL-1*β* and TNF-*α* to experimental animals decreases BBB selectivity. Cytokines induce glial stimulation.

Oligodendrocytes play a central role in the pathogenesis of a wide spectrum of neurological disorders encompassing various neurodegenerative diseases, besides the classical demyelinating disorders. The interaction between oligodendrocytes and other glial cells like microglia offers an insight into the neuroinflammatory dynamics in different neurological conditions. More studies are needed on the communication between microglia and oligodendrocytes. The outcome will help to develop new approaches to treat disorders with myelin damage associated with innate immune activation, promoting repair and reducing inflammation in the CNS. This is summarized in Figure 1.

### 3.3. CNS Immune-Mediated Inflammation, Hypoxia, and Oxidative Stress Crossovers

The CNS is sensitive to peripheral inflammatory events and peripheral immune cell and cytokine infiltration. Unfortunately, unsuccessful repair leads to lasting cellular damage. Any insult to the CNS involves immune-mediated inflammation-hypoxia and oxidative stress. Often, there is a massive epithelial cell loss and interstitial fibroblast proliferation with an extracellular collagenous matrix deposition known as fibrosis because of a failure in repairing injured parenchyma cells [125]. This interpretation is not conclusive. Fibroblast expansion is intrinsic to damage due to tissue-resident macrophage activation and macrophage-like cell influx rather than parenchyma repairing attempt by macrophages. Whether fibrosis benefits or aggravates damage is not clear [126]. Clarifying this issue applies to whether the intervention should point against fibrosis development (fibroblasts' expansion and collagen deposition) or not if the repair strategy avoids axonal loss and brain damage.

Functional recovery after hypoxic brain damage poses a complex scenario. Hypoxia impairs gene expression and downregulates transcription and translation mechanisms and gene activation as the hypoxia-inducible factor (HIF1-*α*) and its target molecules [127]. Hypoxia triggers two main molecular and cell cascades. One leads to hypoxia-damaged cell removal via ubiquitination, peroxisome, and caspase pathway activation, resulting in apoptosis or necrosis, the latter encompassing proinflammatory effects [128, 129]. The other is compensatory, reducing cell loss via multiple mechanisms, including DNA repair, preserving homeostasis [130].

Eventually, the loss and salvage of cells impact brain development, neuronal wiring, and neuron-glia interactions. Whatever further negative impact comes up during development will reinforce the sequel of damage, aggravating neurological deficits and ensuing neurological disorders.

Inflammation and hypoxia are inextricably linked. Nuclear factor kappa B (NF-*κ*B) regulates the HIF1-*α* system [131]. The concept of hypoxia leading to inflammation has gained general acceptance after studies on the hypoxia signaling pathway. Mountain sickness increases the circulating level of proinflammatory cytokines and vascular leakage, triggering pulmonary or cerebral edema [129, 132–134].

**Figure 2:**
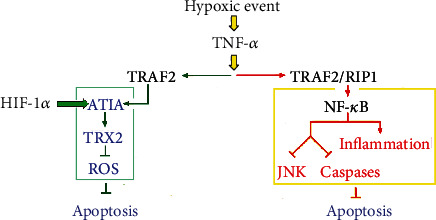
Schematic representation of ATIA as the proposed inflammation and hypoxia crossover.

Hypoxia signaling and the NF-*κ*B family of transcription factors regulate inflammation and orchestrate immune responses to guarantee tissue homeostasis [135]. The interaction of the NF-*κ*B family with the HIF pathway links inflammation with hypoxia. The NF-*κ*B-independent ATIA- (anti-TNF-*α*-induced apoptosis-) thioredoxin 2 (TRX2) axis inhibits TNF-*α*- and hypoxia-induced apoptosis irrespective of NF-*κ*B through TRX2-mediated elimination of excess reactive oxygen species (ROS) (Figure 2) [136].

Ischemia-reperfusion activates NF-*κ*B in epithelial cells, releasing proinflammatory tumor necrosis factor *α* (TNF-*α*) while attenuating apoptotic hypoxia-activated pathways [137, 138].

One study identified an NF-*κ*B-independent ATIA-thioredoxin 2 axis that inhibits TNF-*α*- and hypoxia-induced apoptosis, eliminating ROS directly [139]. Currently, the paradigm for inhibition of TNF-*α*-induced apoptosis points to NF-*κ*B, which inhibits caspases and prevents sustained JNK activation [73]. Besides, the antiapoptotic effect of NF-*κ*B has been associated with excessive ROS elimination.

The evidence poses a novel paradigm for apoptosis inhibition by TNF-*α* and other death signals, controlling ROS accumulation. The pleiotropic inflammatory cytokine TNF-*α* regulates immune responses, inflammation, proliferation, and cell death (apoptosis and necrosis) and regulates apoptosis binding to its membrane receptor 1 (TNF-R1).

Upon TNF-*α* stimulation, TNF-R1 trimer recruits multiple adaptors like TRAF2, TRAF5, RIP1, cIAP1, and cIAP2 and other modulators or regulators like Miz1 and the linear ubiquitin chain assemble complex [140–143].

Cells of the adaptive and innate immune systems in the brain parenchyma and meningeal space are relevant in both brain health and disease.

The ATIA-TRX2 axis inhibits apoptosis induced by both TNF-*α* and a low oxygen level, eliminating excessive ROS in mitochondria. This rescues parenchyma cells from undergoing apoptosis. The activity of ATIA may be a key regulator in carcinogenesis because tumor cells often take advantage of normal tissue under hypoxic conditions.

### 3.4. Autophagy-Associated Inflammation in the CNS

Autophagy plays an important role in both innate and adaptive immune responses [144]. This lysosome-dependent catabolic process serving to the turnover of proteins and organelles is crucial in the inflammatory response and cell survival. Immune and inflammatory signals induce autophagy in macrophages through TLRs, among others [145]. Nevertheless, the physiological role of autophagy and its signaling mechanisms in microglia remains poorly understood [145]. Autophagy-related genes (Atg) in microglia are largely suppressed after TLR4 activation by lipopolysaccharide (LPS), inversely as the LPS-mediated stimulation in macrophages [145].

Microglial cells are activated during various phases of tissue repair in certain CNS pathologies. Spinal cord injury- (SCI-) associated anoxemia has a key pathogenic effect, resulting in tissue damage. Besides, HIF-1*α* protects against apoptosis and necrosis under ischemic and anoxic conditions, upregulating the expression of downstream target genes in brain stroke. Both HIF-1*α* expression and autophagic cell death were described in microglial cells during brain damage [146]. Autophagy suppression with decreased cell viability and increased inflammatory cytokines were reported associated with HIF-1*α* inhibition or HIF-1*α* silencing [146]. If confirmed, HIF-1*α* may lead to minor autophagic cell death of microglial cells associated with hypoxia-mediated inflammation and may provide a novel therapeutic approach for SCI diseases with deleterious microglial cell activation.

Certain bacteria and pathogenic viruses are implicated in neurodegenerative processes, oxidative stress, decreased autophagy, synaptopathy, and neuronal death [147]. However, how infections influence neurological disease progression is still controversial. Mitochondrial antiviral signaling (MAVS) protein has an important role in antiviral immunity and autoimmunity. However, the pathophysiological role of this signaling pathway, especially in the brain, remains elusive [148]. Autophagy regulated MAVS signaling activity in mouse embryonic fibroblasts (MEFs) [149]. In addition, defective autophagy was associated with neurodegenerative disease development [150–152]. Also, MAVS signaling was involved in microglial activation in vivo [148]. Inflammation is concurrent with autophagic activation, and autophagy inhibition in microglial cells strengthens MAVS-mediated inflammation [148]. This accounts for a regulation of MAVS-dependent microglial activation in the CNS, where autophagy has a key role in microglia-driven inflammatory brain diseases.

MicroRNAs (miRNAs) have a role in regulating immune cell development and modulating innate and adaptive immune responses [153]. Abnormal autophagy occurs during infectious and autoimmune diseases associated with certain miRNAs as novel and potent modulators of autophagic activity [154]. The deficiency of miRNA 223 has been found to reduce CNS inflammation, demyelination, and the clinical symptoms of experimental autoimmune encephalomyelitis (EAE) and increased resting microglia and autophagy [154] found that. Taken together, targeting autophagic proteins may be considered as a potential therapeutic strategy in neuroinflammation-associated diseases [144].

### 3.5. Neuroinflammation and Natural Immunity

The innate immune response is the first line of defense after tissue injury, hypoxia, or metabolic stress. Activation of innate immunity in response to tissue injury is crucial to homeostasis restoration and wound healing [155, 156].

**Figure 3:**
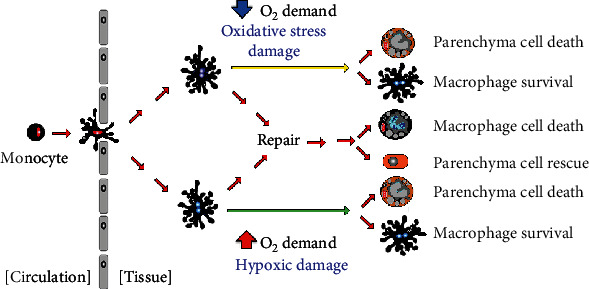
Schematic representation of parenchyma and macrophage cell fate in hypoxia and oxidative stress-inflammation environment.

A balanced oxygen environment is imperative for survival, while away from the balance point, it may be harmful (Figure 3). Both oxygen deficit and excess are detrimental to parenchymal cells and favor macrophage influx [157–159].

Following an insult, cell fate depends on the balance between cell damage and repair, along with oxygen level restoration.

During hypoxia-driven inflammatory damage and oxidative stress-associated inflammatory injury, cell rescue is possible, and parenchyma cells survive. In both scenarios, the immune system orchestrates immune reactive CNS components to restore homeostasis, maximizing parenchyma survival. Provided oxygen level normalizes by homeostatic immune-mediated compensatory mechanisms, parenchyma cells may successfully recover, and the infiltrated macrophages die.

Fully restoring altered homeostasis is not possible in the innate autoimmune response, and inflammation perpetuates [160]. Studies on the interlinkages between hypoxia, tissue alarm signals, neoangiogenesis, and reactive tissue repair mechanisms have allowed identifying early immune response molecules. These are the TLRs, inflammatory cytokines, and putative danger signals, among others, that trigger, sustain, and end the homeostatic response. Janeway's “recognition of microbial nonself” hypothesis explains the activation of an immune response to infection or injury [161]. The “danger model” postulates alternative mechanisms for inducing an appropriate immune response unless there is evidence of tissue injury, termed as “alarm” signals [162]. Innate receptors, like C-type lectins and TLR, seem involved in neuroinflammation and might play a crucial role in the pathogenesis of EAE, an MS animal model [155, 156, 163, 164].

Growing evidence shows that macrophages have various functions in the CNS. Understanding the mechanisms governing the reparative and pathological properties of activated macrophages is at the forefront of neuroscience research. Both macrophage-mediated repair and macrophage-mediated injury occur. Two innate immune receptor subtypes participate in developmental processes and neurological diseases. Danger-associated molecular signals released from dying cells in the injured spinal cord appear to activate different subtypes of macrophage pattern recognition receptors, including TLRs and fungal C-type lectin receptors (e.g., dectin-1) causing neuroprotection or neurotoxicity [165].

**Figure 4:**
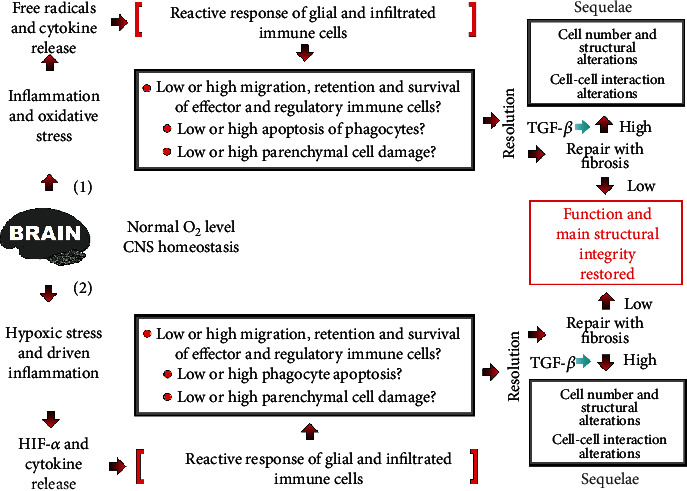
Hypoxic and nonhypoxic inflammation and neuroimmune interactions involved in the prohemostatic response in the CNS. Brain parenchyma, the functional tissue, comprises neurons and glia cells. Brain damage or trauma often leads to cognitive deterioration and/or motor disability with parenchyma structural alterations and eventual cell death. Triggering (1) nonhypoxic and (2) hypoxic reactive inflammation might subserve functional postinjury recovery. Oxidative stress by a high oxygen level induces a compensatory antioxidant response to cut out damage progression. At the other end, hypoxia (hypoxic stress) by a low oxygen level upregulates pathways involved in boosting the oxygen supply. In any case, a fault in oxygen homeostasis draws inflammation with immune cell infiltrates and resident glial cells to restore homeostasis. Light-blue arrow: regulation; red arrow: stimulation.

Oxidative stress and hypoxic stress trigger divergent pathways to restore homeostasis, resulting in survival or death according to the cell type. Hypoxia often amplifies inflammation and has a prosurvival effect on neutrophils, monocytes, and eosinophils. Complete restoring of oxygen homeostasis ensues macrophage apoptosis and wound healing (Figure 4).

### 3.6. Neuroinflammation and Adaptive Immunity

Adaptive immunity makes use of immunological memory to recognize specific pathogens, adding up to the innate immunity response, overall achieving an amplified response. Adaptive immunity is typically initiated after innate immune cells like dendritic cells, macrophages, or microglia via their pattern recognition receptors (PRRs) recognize broad specificities of pathogen-associated molecular patterns (PAMPs) and damage-associated molecular patterns (DAMPs). These are associated with microbial pathogens, cellular stress, or cell components of damaged tissues [166]. In addition, adaptive immunity includes a plethora of effector T cells (Th1, Th17, Th3, Th2, and T regulatory), effector B cells, and antibodies that, in turn, infiltrate the brain during neuroinflammation. Macrophages can act during innate and adaptive immune responses.

During brain hypoxia, the NF-*κ*B pathway is amplified, upregulating TLRs, which enhance antimicrobial factor production and stimulate phagocytosis, leukocyte recruitment, and adaptive immunity. Besides, HIF-1*α* increases, influencing adaptive immunity. Patients with a rheumatic disease showed HIF-1*α*-deficient lymphocytes and high serum levels of anti-double-stranded DNA antibodies and rheumatoid factor [167].

Brain hypoxia depends on signaling mediated by T cell HIF-1*α* receptors [168]. In vivo and in vitro experiments have suggested that immune responses mediated by T cells and HIF-1*α* are key downregulators in vascular inflammation and remodeling tissue, contributing to vascular remodeling modulation together with B lymphocytes [169]. The deletion of HIF-1*α* in T cells impairs differentiation of CD4+ Th17-producing cells in vitro and in MOG/CFA-induced EAE [168]. In a brain hypoxia-ischemia (H/I) mouse model, the expression of TIM-3 (a member of the T cell immunoglobulin that downregulates the TH1-dependent immune response) increases in activated microglia and astrocytes (brain resident immune cells) depending on HIF-1*α* [170]. Blocking of TIM-3 reduces infarct size, neuronal death, edema formation, and neutrophil infiltration in H/I mice [170]. Other studies suggest that HIF-1*α* modulates T cell differentiation towards a Th17 cytokine-secreting phenotype. A decrease in HIF-1*α* resulted in reduced Th17 but enhanced T regulatory cell differentiation, protecting mice from autoimmune neuroinflammation [171]. Others reported that HIF-1*α* induced FoxP3+ Tregs during inflammation [172]. Likewise, in the EAE model, CD4+ cells decreased, and the CD4+CD25+FoxP3 Treg subset increased in the spinal cord of EAE mice exposed to chronic mild hypoxia compared with normoxic counterparts [173]. The increase in Trx-1 contributes to reducing Treg sensitivity to oxidative stress. Along with inflammatory stimuli, especially TNF-*α*, this dynamic negative feedback promotes Tregs in the inflammatory milieu to prevent a sustained or excessive immune response [145, 174]. Inflammatory mediators like cytokines dependent on the Th1, Th17 lymphocyte subpopulation [175], NO, or free radicals [176, 177] have been observed during clinical relapse phases in MS. Conversely, suppressive cellular activity by Th2, Th3, and Tr1 cells, in particular, has been reported during remission periods [178]. The increase in CD4+ and CD8+ T cells found in mouse models of AD [179] suggests an important contribution of T cells to disease pathogenesis [180]. Depleting Tregs enhanced T cell infiltration and reactive astrogliosis in a model of TBI, suggesting tissue damage modulation by Tregs following injury [181].

The evidence of the role of innate and adaptive immunity in neuroinflammation is conclusive. The key events triggering the pathology or charting the chronology of the early changes upon disease is yet to be clarified, even considering the vast literature available. The infiltration of immune cells, T cells, in particular, prompts further examining the role of adaptive immunity [179].

## 4. Fibrotic Reaction to Inflammation

The regulation of fibrotic processes in the CNS is little known. After an inflammatory response, the fibrotic reaction ensues the increase in extracellular matrix components. Different chronic inflammatory diseases with MS-resembling traits like psoriasis or rheumatoid arthritis present severe and intermittent progression with phases of acute exacerbation and remission. They show an influx of inflammatory cells (macrophages, granulocytes, and T cells) and increased expression of proinflammatory mediators, including those locally released by parenchyma cells. These diseases may nevertheless differ in their pathogenesis [182].

Eventually, inflammation rests, but massive fibrosis prevents fully restoring tissue integrity. Even three decades after identifying the master cytokine in immune regulation and fibrosis, we find it hard to ascribe only one role to TGF-*β* [183, 184].

Regulatory T cells (Tregs) release TGF, a potent cytokine that downregulates immune responses and is involved in tissue-specific repair and homeostasis [185].

Most responses to brain injury involve reactive gliosis, resident astrocyte hypertrophy, and neuron cell loss with fibrosis. Fibrosis engages fibrocytes and macrophages derived from the bone marrow. Fibrocytes and activated macrophage type 2- (M2-) microglia cells may act as profibrotic in the CNS as well [186].

**Figure 5:**
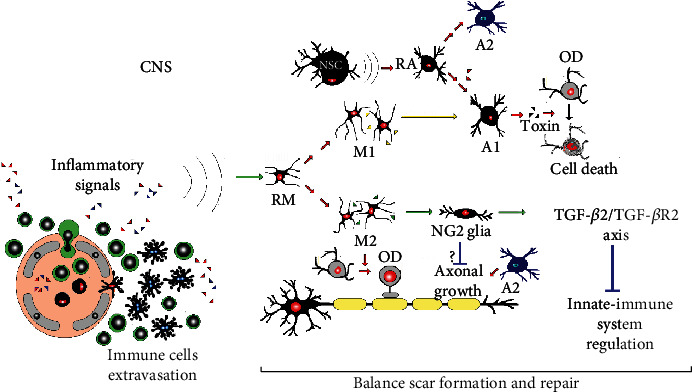
Complexity of brain cell interactions in scar formation and repair.

The glial scar is a structural formation of reactive glia around a damaged area. Traditionally viewed as a hindrance to axon regeneration, beneficial functions of the glial scar have been recently reported. Discrepancies have been discussed on the functional heterogeneity of the glial scar cells, astrocytes, NG2 glia, and microglia (Figure 5). The NG2 glia regulates brain innate immunity via the TGF-*β*2/TGFBR2 axis [187]. After TBI, ischemic stroke, and neurodegenerative diseases, including MS, newly proliferated reactive astrocytes are observed. The NG2 glia and microglia round the severely damaged area or lesion core. This core presents perivascular-derived fibroblasts, pericytes, ependymal cells, and phagocytic macrophages. Previous studies have sometimes referred to the entire CNS lesion as the glial scar, leading to discrepancies. Different glial cells are associated with the lesion or fibrotic lesion core, rich in extracellular matrix proteins, inhibiting axonal growth and remyelination. Yet, some glial cell types counteract, and others regulate scar formation [188].

Immune neuroinflammation involves complex neural and immune cell interactions, regulating the balance between neural tissue repair and scar formation. Reactive microglia (RM) differentiation leads to microglial subpopulations, like macrophage differentiation pathways (inflammatory type M1 and anti-inflammatory type M2). M1 microglia induces A1 reactive astrocyte (RA), derived from a common precursor astrocyte (nervous stem cell abv-NSC-), which under certain signals differentiates to astrocyte cell phenotypes A1 (A1 astroglia are neurotoxic) and A2 (A2 astroglia are neuroprotective). The A1 astrocytes secrete a toxin that kills oligodendrocytes (OD). The A2 astrocytes promote axonal growth. The M2 microglia induces NG2 (neuron-glial antigen 2, also called oligodendrocyte precursor cells) glia differentiation to oligodendrocytes. In addition, NG2 glia regulates brain innate immunity via the TGF-*β*2/TGFB-R2 axis.

## 5. Conclusion

Classifying neuroinflammatory and neuroimmune reactions as beneficial or detrimental is an oversimplification. There is a myriad of interactions between diverse brain cell types and the triggered signaling cascades in different disorders. Various families of cytokines and cytokine receptors, cell-specific distribution, growth factors, and chemokines influence the apoptotic or survival pathways of neurons and the degree of inflammatory processes in the CNS. Even with the growing knowledge of neuroinflammation in health and disease, a deep comprehension of the underlying mechanisms in neuropathology remains limited.

Hypoxia interacts with inflammation at the molecular, cellular, and clinical levels. The immune system reacts to restore homeostasis in two crucial scenarios. One of them is hypoxic stress, causing cells to upregulate pathways involved in increasing oxygen supply. The other one is oxidative stress, causing cells to upregulate antioxidant pathways. Provided that oxygen homeostasis is achieved, epidermal cells survive, and inflammatory leucocytes die. Targeting oxygen-sensing mechanisms and hypoxia signaling pathways might aid in reducing inflammation. Oxidative stress and inflammation underlie most neurological disorders, whether neurodegenerative, autoimmune, traumatic, neoplastic, ischemic, metabolic, toxic, infectious, or other. All of them show direct and indirect immune-related neuroinflammation.

Targeting hypoxia-dependent signaling pathways might help to attenuate organ failure, reducing hypoxia-driven inflammation. Chronic and/or sustained inflammation and hypoxia lead to the survival of macrophages, which further releases oxidative and inflammatory mediators [189, 190].

Inflammatory conditions like meningeal infiltrations, meningoencephalitis with perivascular infiltrates, reactive gliosis, and inflammatory-necrotic lesions showed central immune interactions in different homeostatic alterations.

Regardless of the infective nature, or not, of the central insult, the immune-mediated neuroinflammation orchestrates the response of reactive CNS components to altered homeostasis. Unsuccessful restoration leads to disease, sometimes perpetuating neuroinflammation, and damage. Whether fibrogenesis should be disrupted or not is crucial to understand the pathogenesis and how to go ahead.

There is still a road to walk before a deep insight into underlying factors in pathogenesis allows for designing better treatments.

## Abbreviations

AD: Alzheimer's disease
ADCC: Antibody-dependent cell-mediated cytotoxicity
APC: Antigen-presenting cell
Atg: Autophagy-related gene
ATIA: Anti-TNF-*α*-induced apoptosis
BBB: Blood-brain barrier
bFGF: Basic fibroblast growth factor
BOL: Bovine oligodendrocytes
C3^−/−^: Complement C3 deficient
CCL2: C-C motif ligand 2
CCL3: C-C motif ligand 3
CCR2: Chemokine receptor 2
CD: Cluster of differentiation
CD200: Cluster of differentiation 200
CD4+Th17: CD4+ T helper 17 cells
CD8+ T: Cytotoxic T lymphocytes
ClAP1: Cellular inhibitor of apoptosis protein-1
CLAP2: Cellular inhibitor of apoptosis protein-2
CM: Coccidioidal meningitis
CNS: Central nervous system
COX2: Cyclooxygenase 2
CCR: C-C motif chemokine receptor
CSF: Cerebrospinal fluid
CX3CL1: C-X3-C motif ligand 1
CXC: Cysteine X cysteine
CXCL10: C-X-C motif chemokine ligand 10
CXCL11: C-X-C motif chemokine ligand 11
CXCL12: C-X-C motif chemokine ligand 12
CXCL9: C-X-C motif chemokine ligand 9
CXCR3: C-X-C motif chemokine receptor 3
DAMP: Damage-associated molecular pattern
DNA: Deoxyribonucleic acid
EAE: Experimental autoimmune encephalomyelitis
EBV: Epstein Barr virus
FGF-2: Fibroblast growth factor-2
FoxP3: Forkhead box P3
GPCR: G protein-coupled receptors
H/I: Hypoxia-ischemia
H19-7: Imprinted maternally expressed transcript (nonprotein coding)
HIF1-*α*: Hypoxia-inducible factor-1-alpha
HIV: Human immunodeficiency virus
HIV-tat: Human immunodeficiency virus transactivator of transcription
HPV: Herpes papillomavirus
IFN-*γ*: Interferon-gamma
IL-10: Interleukin-10
IL-18: Interleukin-18
IL-1R1: Interleukin-1 receptor-1
IL-1*β*: Interleukin-1 beta
IL-4: Interleukin-4
IL-6: Interleukin-6
INOS: Inducible nitric oxide synthase
JNK: c-Jun N-terminal kinase
K: Killer
K^+^: Potassium
KA: Kainic acid
LPS: Lipopolysaccharide
LRRK2: Leucine-rich repeat kinase 2
M1: Macrophage type 1 (proinflammatory)
M2: Macrophage type 2 (anti-inflammatory)
MAVS: Mitochondrial antiviral signaling
MCP-1: Monocyte chemoattractant protein-1
M-CSF: Macrophage colony-stimulating factor
MEF: Mouse embryonic fibroblast
MHC-I: Major histocompatibility complex-I
MIP-2: Macrophage inflammatory protein-2
miRNA: MicroRNA
Miz1: Myc-interacting zinc-finger protein 1
MOG/CFA: Myelin oligodendrocyte glycoprotein/ complete Freund's adjuvant
MS: Multiple sclerosis
NF-*κ*B: Nuclear factor kappa B
NG2: Neuron-glial antigen 2
NGF: Nerve growth factor
NK: Natural killer
NO: Nitric oxide
NOD: Nucleotide-binding oligomerization domain-containing protein
OD: Oligodendrocyte
op/op: Osteopetrotic
PAMP: Pathogen-associated molecular pattern
PD: Parkinson's disease
PGS: Prostaglandins
PI3K: Phosphatidylinositol 3-kinase
PLA2: Phospholipase A2
PRR: Pattern recognition receptor
RA: Reactive astrocyte
RAGE: Receptor for advanced glycation end products
RIP1: Receptor-interacting serine/threonine-protein kinase 1
RM: Reactive microglia
ROS: Reactive oxygen species
SCI: Spinal cord injury
SDF-1: Stromal cell-derived factor-1
SK-N-SH: A neuroblastoma cell line displaying epithelial morphology, growing in adherent culture
SN: Substantia nigra
SNCA: Synuclein gene-*α*
SP: Substance P
T cells: A type of lymphocyte
TBI: Traumatic brain injury
TD: Thiamine deficiency
TGF-*β*: Transforming growth factor-*β*
TGF-*β*2/TGFB-R2 axis: Transforming growth factor, beta receptor 2
Th1: T helper 1 cells
Th17: T helper 17 cells
TIM-3: T cell immunoglobulin and mucin-domain containing-3
TLR4: Toll-like receptor 4
TNF: Tumor necrosis factor
TNF-R1: Tumor necrosis factor, receptor 1
TNF-R2: Tumor necrosis factor, receptor 2
TRAF2: Tumor necrosis factor receptor-associated factor 2
TRAF5: Tumor necrosis factor receptor-associated factor 5
Treg: Regulatory T cells
TREM: Triggering receptor expressed on myeloid cells
TRX2: Thioredoxin 2
VIP: Vasointestinal peptide
*γδ* T cells: Gamma delta T cells.
